# Isolation of *Cryptococcus gattii* from Oregon soil and tree bark, 2010–2011

**DOI:** 10.1186/s12866-014-0323-2

**Published:** 2014-12-21

**Authors:** Emilio DeBess, Shawn R Lockhart, Naureen Iqbal, Paul R Cieslak

**Affiliations:** Oregon Health Authority, Public Health Division, 800 NE Oregon St., #772, Portland, OR 97232 USA; Mycotic Diseases Branch, Centers for Disease Control and Prevention, 1600 Clifton Rd, Atlanta, GA 30330 USA

## Abstract

**Background:**

In Oregon, human and animal infections by *C. gattii* were first identified in 2004. *Cryptococcus gattii* is considered to be an emerging non-zoonotic infection affecting animals and humans in Oregon. We report a longitudinal environmental isolation of *C. gattii* after an Oregon dog was diagnosed with the disease in 2009.

**Results:**

*Cryptococcus gattii* was isolated twice from the same location with a span of one year between isolation dates. *Cryptococcus gattii* molecular types VGIIa and VGI were isolated in 2010 from soil and tree bark near the home of a 9-month-old dog which three months previously had an infection caused by *C. gattii* genotype VGIIa. The environment featured heavy growth of Douglas Fir trees. In 2011, a second set of soil and tree bark samples was collected in the same area and *C. gattii* VGIIa was again identified from the environment, along with genotypes VGIIb and VGIIc.

**Conclusions:**

The use of animal surveillance data to identify environmental niches of *C. gattii* should be considered to expand the understanding of this emerging pathogen. Understanding the ecology and how the environment and other factors might modify the existing niches is important for assessing risk and for designing measures to protect human and animal health.

## Background

Infection by the fungus *Cryptococcus* causes pneumonia, skin infection and meningoencephalitis [1-7]. While *C. neoformans* is the most common cause of fatal meningoencephalitis, especially in HIV patients, *C. gattii* has caused disease mainly in non-HIV patients [8]. In 1999, *C. gattii* infections emerged on Vancouver Island, British Columbia (BC). Nearly all infections were caused by *C. gattii* genotype VGII, but the strains lacked the genetic diversity seen among other VGII isolates from other countries and appeared to represent distinct clonal populations, dubbed genotypes VGIIa and VGIIb [9,10]. During subsequent years, *C. gattii* spread to the BC mainland. In Oregon, human and animal infections by *C. gattii* were first identified in 2004, and they have subsequently been identified in Washington State as well [11]. Interestingly, *Cryptococcus gattii* genotype VGIIc, which had not been identified in BC, has been diagnosed in animals and humans in the US Pacific Northwest (PNW) and seems to be centered in Oregon [12,13].

The only known means of acquisition of *C. gattii* infection is inhalation of spores from the environment. Estimation of the incubation period became possible with the identification of a focal reservoir of the fungus on Vancouver Island; an analysis of early cases among residents of the BC mainland with discreet histories of travel to Vancouver Island found incubation periods ranging from 2 to 13 months, with a median of 6–7 months [6,7].

From December 2004 through December 2012, 82 human cases of *C. gattii* infection were reported in Oregon, but early attempts to isolate the fungus from the environment around Oregon case residences were unsuccessful [14]. In 2008, reporting of cryptococcal infection among animals in Oregon became mandatory for veterinarians and veterinary laboratories and 67 animal cases were identified through December 2012 — 28 cats, 16 dogs, 8 alpacas, 7 goats, 3 elk, 2 ferrets, 1 horse, 1 sheep and 1 porpoise. The identification of *C. gattii* infection in domestic animals suggested that exposure to the fungus must have occurred near the animals’ residence and presented opportunity for identifying ecological foci of *C. gattii* in Oregon.

**Figure 1:**
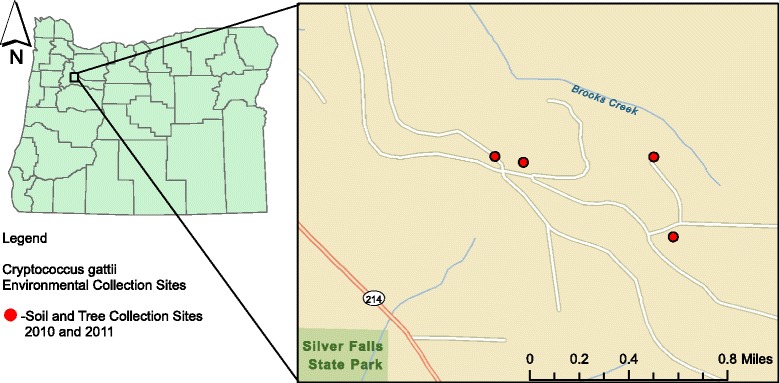
**Geographical location of** ***C. gattii*** **environmental samples, Oregon 2010–2011.**

In March 2010, a 9-month old dog in northwestern Oregon, without travel history, presented to a specialty clinic for examination of periorbital swelling. The animal was in good health except for the swelling. The attending veterinarian obtained a biopsy sample of the periorbital area for cytology and culture. The culture yielded *Cryptococcus*, and the isolate was sent to Oregon State University, Veterinary Diagnostics Laboratory (OSU, VDL) for further identification. It was identified as *C. gattii*, genotype VGIIa. The dog lived in a rural area at 177 feet elevation, heavily populated primarily with Douglas fir trees (*Pseudotsuga menziesii*)*.* The property is located less than 30 feet from an unpaved logging road where several times a day logging trucks carry recently harvested Douglas Firs (Figure 1). This report describes the results of environmental sampling around this case.

## Methods

Environmental sampling was conducted in June 2010 at selected sites and areas surrounding the home environment of the dog with *C. gattii* infection. Sampled environments included areas frequented by the dog, including front and back yards, ditches, a rabbit farm and walking trails. Samples of tree bark (~10 g), woody debris (10–20 g), soil (50 g), and water (50–100 mL) were sampled as described by Kidd et al. [14]. Samples were placed in sterile transport bags and stored in the dark at 4°–10°C pending transfer to the laboratory. Two grams of each sample were suspended in sterile water, and the mixture was vortexed and allowed to settle for 10 minutes. Four 200-μL aliquots of each sample were plated on l-3,4-dihydroxyphenylalanine (DOPA) medium and incubated at 35°C for up to two weeks. Between one and six colonies were picked from each environmental positive sample and all picked colonies were genotyped. *C gattii* was confirmed using l-canavanine-glycine-bromothymol blue (CGB) differential medium [15], and every isolate picked was genotyped using multilocus sequence typing as described [14].

A year later, May 2011, samples of soil and tree bark were again collected in the same area and at an additional location along the logging road.

## Results

Of the 12 samples obtained in 2010 around the home of the positive veterinary case, two yielded *C. gattii*; one soil sample had genotype VGIIa isolates, and another had isolates of both VGIIa and VGI (Table 1). Upon return to the same environs in 2011, *C. gattii* isolates of genotypes VGIIa, VGIIb and VGIIc were identified from the bark and soil samples taken along the logging road bordering the same property. The *C. gattii* VGIIa isolates from soil and bark were indistinguishable from the VGIIa isolate obtained from the dog.

**Table 1:** ***Cryptococcus gattii*** **environmental sampling results in the surroundings of a positive veterinary case, Oregon, 2010–2011**

**Year**	**Genotype**	**Location of sampling**	**Site #**
2010	VGIIa	Canine periorbital abscess	
2010	VGI	Soil, wet ditch by road	1
2010	VGIIa	Soil, rabbit farm	2
2010	Negative	Soil, rabbit farm	2
2010	VGIIa	Soil, dry ditch road	3
2010	Negative	Soil edge of logging road	4
2010	Negative	Soil edge of logging road	4
2011	VGIIa	Soil, dry ditch by road	3
2011	VGIIc	Tree bark, dirt logging road	5
2011	VGIIc	Douglas Fir Tree bark	5
2011	VGIIa	Soil, wet ditch by road	1
2011	VGIIa	Soil, wet ditch by road	1
2011	VGIIa	Soil, wet ditch by road	1
2011	VGIIc	Soil, wet ditch by road	1
2011	VGIIa	Douglas Fir Tree Bark, rabbit farm	2
2011	VGIIa	Douglas Fir Tree Bark, rabbit farm	2
2011	VGIIa	Douglas Fir Tree Bark, rabbit farm	2
2011	VGIIa	Douglas Fir Tree Bark, rabbit farm	2
2011	VGIIa	Douglas Fir Tree Bark, rabbit farm	2
2011	VGIIb	Douglas Fir Tree Bark, rabbit farm	2
2011	Negative	Soil, dry ditch by road	3

## Conclusion

This is the only longitudinal record of *C. gattii* from the environment in Oregon from the environs of a positive case. As was the experience in Vancouver [10], isolation in Oregon was associated with Douglas Fir trees and extends the ecological niche of *C. gattii* in this biogeoclimatic zone southward from BC almost 500 miles to the Willamette Valley of Oregon. In BC, *C. gattii* was generally isolated from acidic soil, and geographic differences in soil pH may influence the extent of colonization reported. *Cryptococcus gattii* soil colonization also was associated with low moisture and low organic carbon contents although one of our positive soil samples came from a standing puddle. The role that trees play in the life cycle of *C. gattii* is not known, but the association of the fungus with decaying wood, especially Douglas Fir in the Pacific Northwest, but also many other species of tree is suggestive of an endophytic existence [14,16].

Perhaps because of the long potential incubation period, during which individual human cases may be exposed to a variety of biogeoclimatic areas, pinpointing environmental niches for *C. gattii* has proven difficult. After extensive sampling around human cases in Australia, Ellis and Pfeiffer reported the first environmental isolation of the fungus in 1990 from eucalyptus trees and noted that the distribution of cases was similar to that of eucalyptus trees [17]. In BC, *C. gattii* has been isolated from a variety of trees and from soil samples in Coastal Douglas Fir and in the Coastal Western Hemlock xeric maritime biogeoclimatic zones. Similar climatic areas are found in Oregon’s Willamette Valley [18]. The *C. gattii* zone may be expanding, accentuated by the increase of logging in the coastal temperate rain forest zone which continues to be widespread, generally beginning in the easily accessible lowlands and working upslope.

Three features are common to all coastal temperate rain forests: proximity to oceans, the presence of mountains, and as a result of the two, high rainfall [19].

Isolating *C. gattii* from the environment has been challenging in other areas of the PNW [14,17] (R. Wohrle, personal communication), perhaps because cases may have been exposed far from their residences making pinpointing areas to sample difficult. This is the first time that four different genotypes (VGI, VGIIa, VGIIb, and VGIIc) have been isolated from the same general environs. It is important to mention that in our experience, if only a single colony had been genotyped from each plate, we may have missed the genotypes VGI, VGIIb and VGIIc, all of which were isolated from the same samples that contained isolates of VGIIa. The diversity of VGIIa isolates from the PNW is so narrow that even isolates from hundreds of miles apart only have on average 12 single nucleotide polymorphisms between them out of 34,000,000 million nucleotides in the genome [20]. With this high level of clonality, it is nearly impossible to determine the source of a single infection as all of the isolates are nearly identical. Whole-genome sequencing on *C. gattii* isolates will better ascertain the natural source and genomic adaptations of the infection in the Pacific Northwest. Based on a recent study VGII study was highly diverse, demonstrating large numbers of mutational and recombinant events. The three dominant subtypes in the Pacific Northwest were low diversity and completely clonal [20].

The use of animal surveillance to identify environmental sources of the disease should be considered to expand understanding of *C. gattii* environmental niches, as they are ultimately the source of animal and human infections. Although rare*, C. gattii* appears to be an emerging pathogen and understanding the ecological niches and how environmental or other factors might modify the existing niches is important for assessing risk and for designing measures to protect human and animal health.

This publication was supported by Cooperative Agreement Number 3U01CI000306 from the Centers for Disease Control and Prevention. Its contents are solely the responsibility of the authors and do not necessarily represent the official views of the Centers for Disease Control and Prevention.

### Ethics statement

No human subjects, human materials, or human data was used.
